# S196. ASSOCIATION BETWEEN INTRACELLULAR INFECTIOUS AGENTS AND SCHIZOPHRENIA IN KOREA

**DOI:** 10.1093/schbul/sby018.983

**Published:** 2018-04-01

**Authors:** Young-Joon Kwon, Won-Myong Bahk, Bo-Hyun Yoon, Duk-In Jon, MoonDoo Kim, Beomwoo Nam, Eunsung Lim, Sung-Yong Park

**Affiliations:** 1 Soonchunhyang University Chunan Hospital; 2 Yeouido St. Mary’s Hospital, The Catholic University of Korea; 3 Naju National Hospital; 4 Hallym University; 5 Jeju National University; 6 Konkuk University; 7 Shinsegae Hospital; 8 Keyo Hospital

## Abstract

**Background:**

A number of studies have reported association between Toxoplasma gondii (T. gondii) and Chlamydia infection and the risk of schizophrenia. The aim of the present study was to compare the prevalence of T. gondii and Chlamydia infection between the schizophrenia and normal control subjects and to compare the clinical features between seropositive and seronegative Korean schizophrenia patients.

**Methods:**

The rate of serum reactivity to T. gondii, Chlamydia trachomatis (C. trachomatis), Chlamydia pneumonia in 96 schizophrenia and 50 control subjects was investigated using enzyme-linked immunosorbent assay and indirect fluorescent antibody technique. The clinical symptoms of the schizophrenia patients were scored with Positive and Negative Syndrome Scale and a comparative analysis was carried out.

**Results:**

A significant positive association between immunoglobulin G (IgG) antibodies to T. gondii and C. trachomatis in schizophrenia was found, and the odds ratio of schizophrenia associated with IgG antibody was found to be 3.22 and 2.86, respectively. The Toxoplasma-seropositive schizophrenia patient had higher score on the negative subscale N1 and N7 and general psychopathology subscale G13, while C. trachomatis-seropositive schizophrenia patient had higher score on the general psychopathology subscale G10.

**Discussion:**

The results from the present study suggest significant association between T. gondii, C. trachomatis infection and schizophrenia. In future, further studies are needed to elucidate the correlation between the two types of infection and schizophrenia.

